# Designing an intervention to help people with colorectal adenomas reduce their intake of red and processed meat and increase their levels of physical activity: a qualitative study

**DOI:** 10.1186/1471-2407-12-255

**Published:** 2012-06-18

**Authors:** George Dowswell, Angela Ryan, Aliki Taylor, Amanda Daley, Nick Freemantle, Matthew Brookes, Janet Jones, Richard Haslop, Chloe Grimmett, Kar-Keung Cheng, Wilson Sue

**Affiliations:** 1 Department of Primary Care and General Practice, University of Birmingham, Edgbaston, Birmingham, B15 2TT, UK; 2 Amgen Ltd, 1 Uxbridge Business park, Sanderson Road, Uxbridge, UB8 1DH, UK; 3 Department of Primary Care and Population Health, Medical School (Royal Free Hospital), University College London, Rowland Hill Street, London, NW3 2PF, UK; 4 Macmillan Survivorship Research Group, University of Southampton, University Road, Southampton, SO17 1BJ, UK; 5 Royal Wolverhampton Hospitals NHS Trust, Wednesfield Road, Wolverhampton, WV10 0QP, UK; 6 University Hospitals NHS Foundation Trust, Queen Elizabeth Hospital, Mindelsohn Way, Edgbaston, Birmingham, B15 2WB, UK

**Keywords:** Adenomatous polyps, Feasibility studies, Prevention & control, Health behaviour, Attitude to health, Qualitative research, Colorectal neoplasms

## Abstract

**Background:**

Most cases of colorectal cancer (CRC) arise from adenomatous polyps and malignant potential is greatest in high risk adenomas. There is convincing observational evidence that red and processed meat increase the risk of CRC and that higher levels of physical activity reduce the risk. However, no definitive randomised trial has demonstrated the benefit of behaviour change on reducing polyp recurrence and no consistent advice is currently offered to minimise patient risk. This qualitative study aimed to assess patients’ preferences for dietary and physical activity interventions and ensure their appropriate and acceptable delivery to inform a feasibility trial.

**Methods:**

Patients aged 60–74 included in the National Health Service Bowel Cancer Screening Programme (NHSBCSP) were selected from a patient tracking database. After a positive faecal occult blood test (FOBt), all had been diagnosed with an intermediate or high risk adenoma (I/HRA) at colonoscopy between April 2008 and April 2010. Interested patients and their partners were invited to attend a focus group or interview in July 2010. A topic guide, informed by the objectives of the study, was used. A thematic analysis was conducted in which transcripts were examined to ensure that all occurrences of each theme had been accounted for and compared.

**Results:**

Two main themes emerged from the focus groups: a) experiences of having polyps and b) changing behaviour. Participants had not associated polyp removal with colorectal cancer and most did not remember being given any information or advice relating to this at the time. Heterogeneity of existing diet and physical activity levels was noted. There was a lack of readiness to change behaviour in many people in the target population.

**Conclusions:**

This study has confirmed and amplified recently published factors involved in developing interventions to change dietary and physical activity behaviour in this population. The need to tailor the intervention to individuals, the lack of knowledge about the aetiology of colon cancer and the lack of motivation to change behaviour are critical factors.

**Trial registration:**

Current Controlled Trials ISRCTN03320951

## Background

Colorectal cancer (CRC) is the third most common cancer and second most common cause of cancer death in the UK [1]. Most cases of CRC arise from adenomatous polyps and malignant potential is greatest in high risk adenomas [2]. A potentially large increase in the detection of intermediate and high risk adenomas (I/HRAs) is anticipated now that the NHS Bowel Cancer Screening Programme (NHSBCSP) has been implemented nationally in the UK [3]. However, no consistent advice is offered to these patients in order to minimise risk of polyp recurrence, which is estimated to approach 40% at three years [4].

Evidence from the EPIC (European Prospective Investigation into Cancer and Nutrition) study carried out in 10 European countries on 478,040 men and women demonstrated a significant increase in risk of CRC with high consumption of red and processed meat [5]. Results from this study also suggest a significant reduction in the risk of CRC for those with a high dietary intake of fish [5]. Meta-analyses have further confirmed the association between CRC and a high intake of red meat and processed meat [6-8]. Based on a summary of all existing observational studies, the World Cancer Research Fund (WCRF) concluded that there is convincing evidence that red and processed meat increase the risk of CRC and that higher levels of physical activity reduce the risk of colorectal cancer [9], although for physical activity the evidence is less clear for rectal than it is for colon cancer [10]. The WCRF recommends avoiding processed meat in the diet and limiting red meat consumption as well as being physically active in everyday life [10]. Specifically, the recommendations from WCRF for individuals are to consume less than 500g of red meat per week with very little (if any) to be processed meat. WCRF also recommends aiming for at least 60 min of moderate, or at least 30 minutes of vigorous physical activity per day. Since 1996, the English government advice has consistently been to do at least 30 minutes of moderate physical activity five times a week for general health [11].

However, given these recommendations are based on observational studies that merely show an association between diet, physical activity and CRC it is unclear whether behavioural change would result in a reduced incidence of the disease. Therefore, a prospective randomised controlled trial (RCT) is required to confirm the relevance of the WCRF lifestyle recommendations for CRC prevention and satisfy the need for more definitive evidence on the effect of meat and physical activity on risk of CRC. The design and evaluation of complex interventions requires that extensive planning should precede definitive research [12]. There is a need to understand the problem before designing interventions. Randomised trials can then determine whether the anticipated effects from an intervention are in fact delivered in practice. This is a growing field of academic interest, as spiralling health costs are bringing a greater focus on preventive medicine and lifestyle programmes are increasingly being designed for not only cancer prevention [13] but for various populations in different settings [14].

We have long known that human behaviour, health and longevity are related [15]. The benefits of increased physical activity and dietary improvement are not restricted to CRC prevention studies, but also relevant for other cancers, diabetes and cardiovascular disease. Behaviour change has featured strongly as an objective in healthcare [16]. For example, the Theory of Planned Behaviour (TPB) [17] has been used extensively. It suggests that behavioural intentions are dependent upon attitudes towards behaviour, subjective norms and perceived behavioural control. These were issues we wished to explore in relation to planned diet and physical activity related behaviour change.

Previous evaluations of behaviour change strategies have been hampered by poor research designs, the difficulty of separating the effects of different components in complex interventions, and lack of detail in research reports [18]. This prompted us to consider exploratory research with potential participants, local service providers and academics prior to the development and implementation of our intervention. Meetings with local Healthy Lifestyle services and project team members suggested that current health policy on health promotion in England is underpinned strongly by Social Cognitive Theory (SCT) [19]. This emphasises a number of issues, including self efficacy, increasing confidence to make changes, smart goal setting, expectations management (to a more realistic level) and self monitoring (increasing self awareness). It has elements in common with the trans-theoretical model of behaviour change (TTM) [20]. Social Cognitive Theory has more recently been supplemented by Self Regulation Theory (SRT) which includes a “feedback loop” – with evaluation (self or external) in which barriers are identified, social support mobilised and goals are amended upwards or downwards in line with feedback [21]. There are many domains or dimensions which may facilitate or obstruct change but the importance of beliefs about the need to change and the centrality of memory/attention/decision processes in making new behaviour routine have been singled out as particularly important for the design of interventions [18].

This qualitative study aimed to assess patients’ preferences for appropriate and acceptible dietary and physical activity interventions. In order to do this, we sought to refine delivery of planned interventions in the light of identified facilitators and barriers to participation in this patient population. The results of this qualitative study were intended to inform the design of an intervention to enable patients at risk of further I/HRAs to reduce their dietary red meat intake and avoid processed meat and/or increase physical activity levels. In turn, this will inform a definitive RCT to determine whether these interventions can in fact reduce the recurrence of I/HRAs.

## Methods

### Study population

The National Information Governance Board (NIGB) restricts access to patient records for research purposes. For this reason, we had to rely on local clinical collaborators to identify eligible patients and to send the initial communications relating to the study. When a sampling frame had been constructed (258 patients), alternative sampling (every other person) was used to send invitation letters and patient information sheets were sent to half the people aged 60–74 years who had been diagnosed with an I/HRA in each month between April 2008 and April 2010 and included in the National Health Service Bowel Cancer Screening Programme (NHSBCSP). These patients were selected from the Royal Wolverhampton Hospitals NHS Trust patient tracking database and had been diagnosed with a I/HRA at colonoscopy after a positive faecal occult blood test (FOBt). The letters advised patients that partners would also be welcome at focus groups or interviews. A reply slip with a prepaid envelope was enclosed to indicate if the person and their partner would be interested in participating. Those who were willing to take part were contacted by telephone. A reminder letter was sent after two weeks if no reply was received from the initial letter.

Following the focus groups, a short diet and physical activity preferences questionnaire was sent to the remaining cohort of people aged 60–74 years who have been diagnosed with an I/HRA in each month between April 2008 and April 2010. This was to determine if focus group responses were representative of the wider population. Although it is standard research practice, non-responders to the initial mailing were not sent a reminder because local research management and governance permissions did not allow this.

### Conduct of focus groups and interviews

**Figure 1:**
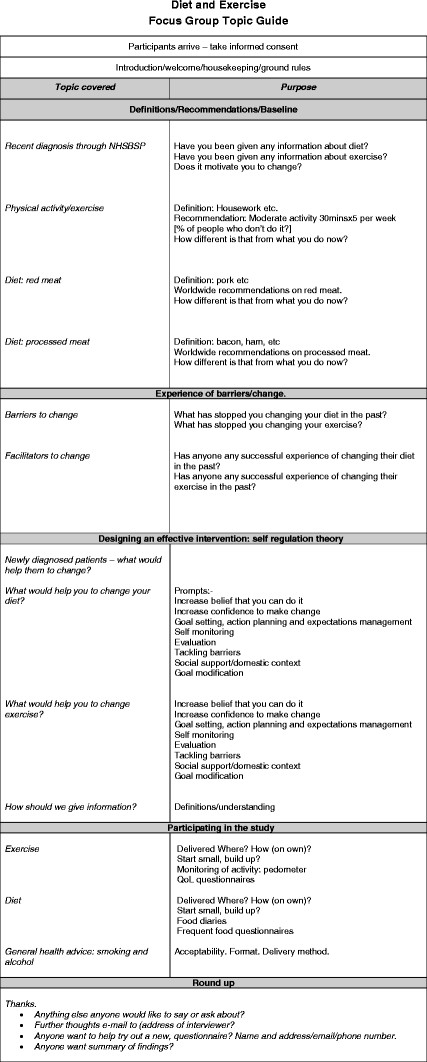
Diet and Exercise Focus Group Topic Guide.

Focus groups were facilitated by GD. Interviews were conducted (by GD or AR) with patients who were not able or willing to participate in focus groups. Written informed consent was obtained from each participant before each interview or focus group and a topic guide, informed by the objectives of the study and reflecting the current knowledge of behavioural change models [22], was used (Figure 1) [23].

### Method of analysis

A thematic analysis of the transcripts was conducted. This involved grouping the data into themes and examining all transcripts to ensure that all occurrences of each theme had been accounted for and compared [24,25]. The transcripts were read a number of times to ensure familiarisation with the data. An initial list of codes was then generated and grouped into defined themes and sub-themes, which were then reviewed, refined and transcripts were labelled or ‘indexed’ [26,27].

## Results

### Study population

**Figure 2:**
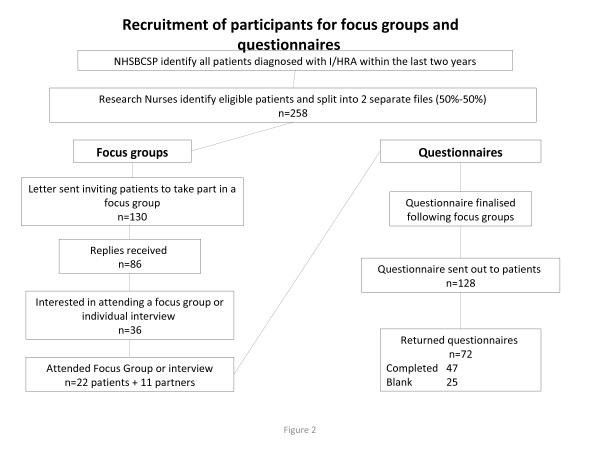
Recruitment of participants for focus groups and questionnaires.

Of the 130 patients invited to participate, 86 (66%) replied; of these 28 (33%) expressed an interest in attending a focus group. Four focus groups were held involving 18 patients (12 men and six women) and nine partners (all women); median number of participants 8 (range 3–8). The mean age of patients who participated was 69 years (SD 4.4; range 60–75 years). Ten patients who had expressed an interest did not take part: three were unable to get to the venue for the focus group, three changed their minds when contacted or after agreeing to attend a focus group, two could not attend on the dates and/or at the times of the focus groups, one was un-contactable by telephone and one could not attend for personal reasons. A further eight patients requested interviews at home: four were contacted and all of them (all men) were interviewed along with two of their partners (both women) (Figure 2).

### Emerging themes

The findings from the analysis can be organised around two main themes (A and B), which can themselves be split into sub-themes. The first theme, A, “experiences of having polyps”, is split into two sub-themes related to participants’ accounts of being diagnosed with polyps: (i) information and advice that participants remember receiving at the time of diagnosis and (ii) beliefs about the general causes of polyps and specifically why they developed them. The second theme, B, called “changing behaviour”, is split into three sub-themes related to participants’ views about and attitudes to changing their diet and physical activity levels: the sub-themes are (i) current behaviour (ii) facilitators for change and (iii) barriers to change.

The emerging themes, sub-themes and relationships are described below. Selected quotations have been used to illustrate the points being made. Given the small number of participants, it is not appropriate or useful to represent these findings numerically [28], although, where it is meaningful, indication has been given to whether the point under discussion was made by the majority or minority of participants.

A Experiences of having polyps

In common with most qualitative studies, participants in focus groups and interviews prefaced their discussion of the principal topics (diet and exercise) by providing background narrative history and perceptual context. Accounts of diagnosis and treatment were shared. Similarities and differences in experience and interpretation were noted. We have not reported most of the similarities in detail (issues of space and interest) but have selected some of the interesting differences in accounts for comparative purposes.

(i) Advice and information

Participants had generally not considered having a polyp removed to be serious and, more specifically, had not associated having a polyp removed with CRC. If, during or after their colonoscopy, CRC had been mentioned, participants had tended to simply feel relieved that they did not have CRC. They had not tended to associate their polyp with any future risk of CRC or think about how they might change their behaviour to reduce their future risk. They were, therefore, usually surprised when they received an invitation to take part in a study related to preventing CRC.

"I think also you don't, I didn't, realise the seriousness of it. You go through the test, you go through the chat at the clinic, you come here and have it done and at the end of the day they say we've removed a polyp and yes it could have been bad and whatever but thank you very much. And you don't come away thinking crikey, that was close. You don't realise the seriousness of it, well I didn't anyway. You just come away and say oh thank you very much, that's great. Focus group 4 patient 1:"

Participants generally did not remember being given any information or advice at the time of their colonoscopy about what polyps are, what causes them and what can be done to prevent further polyps.

"How do these develop? Nobody explains how they develop. Focus group 1 patient 1."

"Well the point is, the only time I went to the hospital was when I went to have it done…. But actually, about diet and things like that, I didn’t know anything about it until you’ve just mentioned that. Interview 2 patient"

Some people did remember receiving information leaflets, but they could not remember the exact information that they contained or did not find this information useful. Only one person specifically said that they remembered receiving information about reducing red or processed meat or increasing physical activity levels.

"I had a leaflet which said eating fruit may help but I wasn’t encouraged. Nothing was really explained. Focus group 1 patient 2"

"Yes, I had a little booklet and it tells you there different things, that red meat was one of the main reasons it causes them. Interview 1 patient"

Most participants were interested in receiving more information about polyps and would have appreciated advice around this time, although some felt that they were too drowsy (following anaesthetic) or concerned (by hospital procedure) to take in advice actually on the day.

"I think we need to know really what causes a polyp to start. Is it a weakness in the bowel that develops it? Focus group 3 patient 4"

"What I would really like to know is what is a polyp? What is it made of? Focus group 3 patient 1"

(ii) Beliefs about the causes of polyps

In the absence of concrete information, some participants had constructed their own complex arguments for why they had developed polyps. One person, for example, related her diagnosis to a combination of falls and her family history of the condition.

"I've had two falls basically. I had a haematoma of the buttock about five years ago, maybe six years ago. And then I fell again and I broke, I had a dislocation of the ankle and pins in place in my foot. And each time when I fell, it's on that right buttock. And I think it's all come from that. Focus group 3 patient 1"

"I've got a brother who is 80 now and he's got exactly the same as I have and he's been having it longer than me. So this is why I think it's genetic. It's in the family. Focus group 3 patient 1"

Another participant talked about how polyps are common, something that many people are just likely to get at some point in their lives and hence not something to be unduly concerned about.

"Well I didn’t give it a great deal of thought to be honest because I understand polyps are quite common, it’s not terribly uncommon so it didn’t alarm me a great deal to be honest with you. Interview 3 patient"

Some participants or their partners felt that getting a polyp was unpredictable, without necessarily a particular cause. Participants illustrated this by describing how some people follow all the rules but still get polyps, whereas others do the opposite and remain healthy.

"Well first of all it didn't occur to me that it was diet related. I mean I'd got to 72, I'd never been ill and so I just thought it was one of those things that happen. Focus group 1 patient 4"

"We're both on the same diet and I've had two colonoscopies and I haven't got polyps. You're predisposed to it, that's my view. Focus group 3 partner 4"

"Well I know people that have beef every day of the week and they never get anything wrong with them. I'm not kidding you. Interview 4 patient"

B Changing behaviour

(i) Current behaviour

Many participants felt that their physical activity levels and/or diet were already very good (specifically if their level of consumption of red meat was already low) although they usually did not rule out further improvements.

"Speaking about the exercise part of it, like this gentleman here, I do a lot of exercise, bike riding. Me and the wife, we think nothing of walking four or five miles a day so I was quite surprised at how big the polyp was…. Focus group 1 patient 2"

"Oh yeah, I’ve cut it tremendously down, even in the week. The only time we really eat red meat is if we have a Sunday lunch. Interview 2 patient"

(ii) Facilitators for change

Many participants described the opportunities provided by retirement, and the lifting of constraints associated with work. Some gained additional physical activity through new responsibilities they had acquired on retirement, for example, looking after grandchildren.

"But my problem is I can walk now because I've just retired but for the last ten years I've been working nights. So when I'm awake my wife’s asleep and the other way round, we only ever used to meet at weekends. So we’d perhaps slot one walk in but for ten years we hardly did anything at all. And I put on weight and ate some horrible food and now my life is changing so we're back to walking again now. Focus group 4 patient 1"

"But it's time with me more than anything else. I'm retired. When I was working I did have a rest when I was at work. [laughter] Don't get any rest now. Focus group 1 patient 4"

Many participants talked about what had already worked for them in terms of changing their behaviour or what they anticipated would work in the future. Some participants felt, in contrast to other people, that they had control over their health and could make any changes that were recommended to them.

"No that is the way I am. I don't think everybody is the same because when I talk to other diabetic people they think I am a different person. I can sacrifice anything, I am born Buddhist, and we learn to practice mind and [it’s] very easy. Focus group 4 patient 3"

"I could stop eating sausages, it wouldn’t matter to me because it’s just something different, it’s not as if I rely on a sausage. Interview 2 patient"

"It would be difficult for me to cut down on processed meats I think but I would have to, if I made an effort I would find ways. Focus group 1 patient 3"

Participants who had managed to change their behaviour in the past tended to relate this to the seriousness of the condition that led to the change, for example that it was life-threatening. Similar to this, other participants felt that this might be the only way to get people to change their behaviour.

"They told me I would lose my leg. That bad. And I'd got 18 cigarettes in the packet. I walked out and I threw them in the waste bin and never smoked since. Focus group 3 patient 3"

"…and they said please get on the couch so I did and she [nurse] went to the phone and said “Doctor, he's ready to have an heart attack, I've never seen someone with such high blood pressure. Focus group 1 patient 2"

"I think if somebody said to you, 'you've got to eat eggs else you're going to die,' you'd eat eggs, wouldn't you? So that's what I mean. Focus group 3 patient 4"

"The only way to stop it is to have a cancer scare, isn't it? Focus group 3 patient 4"

Some participants who had made changes also related this to a consultant giving specific advice. Similarly, other participants felt that change was more likely if recommendations came from trusted sources, such as health professionals.

"I originally started to think about changing my diet, as I said, when I first started with haemorrhoids and I had to see a consultant. He talked through my diet which meant I started to put more fruit and veg in and therefore [husband] had more fruit and veg because I was preparing it all. Focus group 2 partner 4"

"It’s more authoritative if it’s come from the hospital. Focus group 2 patient 2"

"Now, going back to the bowel side of it, I suppose if a professional person, a GP or a consultant even, read the riot act about it, you would seriously think about doing it. Focus group 3 patient 2"

Participants who had managed to change their behaviours, however, also described immediate benefits as being an important reinforcement for maintaining changes. People who had increased their activity levels and/or lost weight, for example, were pleased that they felt better and could do more.

"Yes, one of the things that I've done is when I was trying to keep up with the grandson and I found out I was having a bit of trouble keeping up with him I did go to the gym and I found using the machines in a gym was beneficial. Got more energy. More lung capacity. And I felt a lot better. Focus group 1 patient 1"

"Yes, yeah, because I am overweight, I know that. And when I have lost a bit I've felt a lot better. Focus group 4 patient 1"

In contrast, for some participants, enjoyment of the activity was what was important in maintaining their participation.

"It's just part of my lifestyle. If I had to, at my age, if I had to train specifically for it I'd begin to question whether I was enjoying it any longer. I do it because I enjoy it. And my maxim is as soon as I stop enjoying it I shall stop doing it. It’s as simple as that. I enjoy the company, I enjoy the walks. Focus group 1 patient 4"

Some participants expressed a desire for support from others who had been in a similar situation. Several reasons were given for this: the positive example of someone who had succeeded in making changes and then being effectively cured, the social aspect of meeting other people, the obligation inherent in making an appointment to see other people, and the opportunity to learn from other people.

"You can't convince people - you should have somebody who had polyps once and then it's clear, if that person is on a diet and follows what this person says, once I had in 2008 a few polyps and then when I had three years afterwards my test is clear. That person should be the example for talking to the team. Focus group 4 patient 3"

"I personally would like the discussion group, with somebody like yourself to lead it and then you just listen to ordinary people and their views and you go away and decide for yourself. Interview 3 partner"

Not everyone felt, however, that peer support or discussion groups would be useful for them, highlighting the need for any intervention to be adaptable to each individual’s needs.

"There’s only one thing with that, I’m a loner and my problems I wouldn’t discuss with anyone else apart from my doctor or the wife or family and that’s it. Interview 1 patient"

Finally, thinking about how an intervention should be designed to best encourage people to make changes to their diet and physical activity patterns, one person described how clear descriptions and definitions were necessary and another emphasised the need for short-term achievable goals in order to achieve long-term changes.

"Yes I do, but what would help would be what is meant by processed food. Focus group 3 patient 1"

"They’ve got to get success, you’ve got to give them steps where they can succeed, they’ve got to have challenging targets but achievable targets. It’s no use turning round to people and saying ‘right, by Friday, you’re giving up all red meat, forget that, it’s not going to be there’. Focus group 2 patient 3"

(iii) Barriers to change

Participants talked about what had been and what might be barriers to changing behaviours. These can be divided into two broad groups: those that are related to convincing people about the need for change and those that are related to practical issues associated with actually changing behaviour. In terms of convincing people about the need for change, some participants simply did not want to know about possible causes of illness, saying instead that ignorance is bliss.

"And I don't ever remember, until I came here to have this colonoscopy, I had my appendix out when I was about 43, never had a day’s illness in my life before. I worked 30 years in the steel industry, never had a day off with illness, so it was a real shock and I think I come from the school where ignorance is bliss occasionally: the more you get to know about things that are supposed to harm you, the more they harm you, which is quite worrying really. Focus group 1 patient 4"

Several participants (8/33) identified the lack of any evidence that was good enough and not just “statistics” to convince them that this change was really necessary.

"It's like all these statistical surveys that are done. They produce some results which are totally wrong in many cases just because 50 people in the survey developed polyps because they sat down, there's 500 who sit down who didn't develop them in another survey. So I was getting at, is there a medical reason for the results? Focus group 3 patient 4"

In line with this, many participants identified the issue of confusing and conflicting evidence from many sources, for example that a foodstuff can be reported as good for you one day and harmful the next day. This also tended to be mentioned by partners who may be more involved in buying foods and planning menus.

"Yes and if it is consistent advice because I think most of us will be back every year for a check up and so on. If it is consistent and we don’t suddenly find out that this year is totally different from last year’s advice, then we just become sceptical again. Focus group 2 patient 1"

"The other thing is you get so differing things all the time. One minute they say in the paper you mustn't have tea and then the next day, tea is fantastic for you. I'm only giving you an example. I follow it all because of having had breast cancer and I thought I shouldn't have milk and dairy so I'm having soya. And now I've seen another thing saying soya's not good for you. So you just don't know what to do. Focus group 3 partner 4"

Some participants (4/33) also talked about the difficulty of providing convincing evidence on an individual basis that any changes that had been made had been beneficial. This was partly a recognition that outcomes may be several years after any behaviour change.

"I think it’s difficult to say whether any changes you make will affect how you feel because you can’t compare it with how you would feel if you hadn’t done X, Y or Z. Interview 3 patient"

"So I think the problem you’ve got with a polyp is with cholesterol you’ve got a figure you can aim for. With a polyp because you don’t know what’s going on there, you’ve got no way of really knowing if your change of diet is really doing anything anyway. Focus group 2 patient 2"

Participants tended to express scepticism that one solution would suit everyone

"We’ve also got a very good friend who eats with us a lot but their cholesterol readings were down at three point something. Mine, who I hardly have any milk, hardly have any butter, mine was nine point something and yet my diet, according to the rating for cholesterol, was far, far better than either of theirs. In fact, we were absolutely shocked when my cholesterol was high but theirs was down. So is there not anything in your mind to think that maybe our bodies will process different sorts of foods in different ways and do some of us some harm and others not? Focus group 2 partner 4"

"I think everybody’s different - body wise as well aren’t they? I mean different tablets, one does for one doesn’t do for the other. So basically you could say that again for what you eat. Interview 2 patient"

Participants also talked about several more practical issues associated with changing their diet. They described an inertia and lack of interest in changing their behaviour arising from simply being comfortable with the current situation and, similar to this, several people identified enjoyment of certain foods as a reason for maintaining the status quo.

"A lot of people are comfortable with the way their diet is at the moment. I think that’s the big barrier to change, because we’re all comfortable. Focus group 2 patient 2"

"Don't tell us not to eat meat. We like our meat. Focus group 3 partner 2"

"Well I can do without meat up to a point but there’s no way I could do without sausages, I like my sausages you see. Interview 1 patient"

Some put this more strongly, saying that changing their diet would be undesirable because they derived pleasure from eating certain foods and that overly restricting themselves would lead to a life devoid of enjoyment, which would simply be an existence.

"As a bit of treat to yourself because there’s a difference between living and existing and that’s where I think you’ve got to draw the line. Focus group 2 partner 3"

"I mean I think of a friend of ours who’s just recently died in very tragic circumstances. They had a wonderful good diet, very varied, didn’t drink, didn’t smoke, played hockey. He had a major stroke when he was 59. Focus group 2 partner 4"

A number of participants (5/33) described more concrete problems associated with changing their diets. Several felt, for example, that it would be hard to identify possible alternatives to their current favourite foods, particularly processed meats like ham.

"So basically, my diet that I eat now, which like I say I’m not a big eater, if I stopped eating the sausage, bacon, ham, red meat I don’t eat a lot anyway, most probably toast, I don’t know what else I’d eat. Focus group 2 patient 2"

"The saving grace is I love eggs and therefore I can substitute eggs for a lot of things, but it’s finding alternatives.**….** I would almost want specific recipes as alternatives. Focus group 2 patient 3"

Being able to change one’s diet was also not simply in the hands of the patient. Partners who did the cooking appeared to have a pivotal role in changing patients’ diet and they therefore, also needed to be able to identify alternatives to foods that should be cut out or reduced. Also related to the practical difficulties of changing an established diet, one participant mentioned that they simply did not know how to cook anything other than red meat.

"Limitation in my cooking ability [laughs]. I can grill a chop or make a stew or mince and things like that. Focus group 2 patient 3"

As well as discussing problems associated with dietary changes, participants also talked about the practical aspects of changing their activity levels. Although most were retired, many recalled that they would have found it difficult to eat a healthy diet at work, particularly when working irregular hours, or fit in any physical activity, particularly when they were doing a sedentary job. Again on a practical level, many participants felt that other medical conditions made it difficult to change particularly their physical activity patterns.

"Well I used to go to Weight Watchers and they were coming up with different things and all things like that, and then I had arthritis in my shoulders and my legs, so that stopped me exercising. I mean I've had the one done now, I'm waiting for me other one. Interview 4 patient 3"

One participant described a fear of going out at night as a reason for not exercising.

"I'll tell you one reason I don't walk, I'm terrified to go out at night. Focus group 1 partner 1"

The negative impact of a limited budget on the ability to make changes without external assistance was also discussed, although one participant described how more restricted finances had actually facilitated cutting out red meat.

"But the problem with gyms is the fact they're expensive. And you need to go twice week, in the end it's a lot of money. Focus group 1 patient 1"

"I finished work in 2004 and I found my circumstances did change my diet because when I was working, I used to eat a hell of a lot of red meat. When I stopped working, I went onto a pension and my financial situation changed if you like so I was forced by that situation to change diet so that’s why the red meat went out mostly. I found that easy to change and move onto chicken and fish. Focus group 2 patient 2"

### Designing the interventions: questionnaires

We mailed 128 questionnaires and received 47 replies (37% response rate). The mean age of respondents was 67 (SD 4.3: range 61–78) and 32/47 were male (68%), (Table 1 - preferences for delivery of diet and physical activity intervention). Although the response rate was low, the gender balance appeared similar to the population of people with a positive faecal occult blood test (FOBt) identified by the NHSBCSP who have intermediate or high risk adenomas (31.5% of men with + ive FOBt have I/HRA and 20.8% of women) [29].

**Table 1:** Designing the intervention

**Preferences for the delivery of a diet and physical activity intervention**				
			**Health Trainer n = 47 n (%)**	**Dietician n = 47 n (%)**
**Professional contact**	How often	On demand	25 (53)	27 (57)
		Monthly/fortnightly	19 (40)	19 (40)
		Never	3 (6)	1 (2)
	Contact preference	Phone	23 (49)	24 (51)
		Face to face	14 (30)	13 (28)
		Email	7 (15)	9 (19)
**Peer group contact**	Contact preference	None	28 (60)	28 (60)
		Face to face	15 (32)	15 (32)
		Chatroom	4 (8)	4 (8)

More than half of the respondents wanted a flexible, responsive service for both diet (57%) and physical activity (53%). Around half were happy for a telephone based intervention to be delivered. A few people (8%) wanted an internet chat-room where they could interact with others and a third wanted to meet others facing similar circumstances, but 60% did not want any contact with their peers. Almost everyone was willing to record their diet (98%), most wanted printed booklets or leaflets (92%) and most thought a personal profile of progress would be helpful (79%). All times of day and week were possible for at least some respondents but daytime during the working week was acceptable to 72% of respondents. Two thirds of respondents were interested in taking part but a third gave reasons why they would not be interested. These included barriers to participation (15% - such as employment, carer responsibilities, morbidity or transport difficulties) lack of relevance (13% - already knowing recommended behaviours or already following them) and the need for more information before committing to the study (4%).

## Discussion

The main findings were that participants placed their brief experience of polyp removal within their long term narrative life history. There was a general lack of readiness to change (pre-contemplation stage in TTM) in many people in our target population. This was either because they believed their current behaviour was adequate or they perceived no risk to health. Almost all participants reported that they lacked information and hence an awareness of cancer risk, disbelieved evidence they had received and disputed the link between behaviour and outcome. Facilitators for change included opportunities provided by retirement, beliefs in self-efficacy (partly associated with previous successful changes) and clear authoritative guidance. Immediate benefits (health or social), social support and achievable goals were perceived to be important. Barriers to change were both psychological and practical and included lack of motivation, bewilderment from contradictory public health messages, perceptions of a long-term delay between change and ultimate outcome, and widespread observation of idiosyncrasy and variability within human health. Further practical obstacles included satisfaction with current status quo, lack of acceptable dietary alternatives, physical disabilities impeding physical activity and the perceived costs of change. These findings generally confirm and amplify the main results of a similar study recently published [30]. Stead *et al.* found that people who have had adenomas removed lacked perceptions of i) the link between adenoma and cancer, ii) links between lifestyle factors and adenoma and iii) understanding of the causes of adenoma. They also found that reassurance provided by clinicians (‘all clear message’) undermined subsequent prevention messages and increased the difficulty of convincing sceptical people of the need for lifestyle change. All these views were found in our focus group participants.

Previous qualitative work has found that information giving by all national cancer screening programmes (cervical, colorectal, breast) may be inadequate or confusing. This may be because it does not acknowledge cultural differences or is based on erroneous assumptions about the levels and nature of public knowledge and understanding [31-33]. It is also likely that screening clinicians focus on reassuring patients rather than increasing their health awareness. This may stem from relief on the part of clinicians that more serious pathology has not been detected and perhaps may also be perceived by clinicians as an opportunity to send patients away happy. From our local and small scale study, it appears that the opportunity to inform patients about I/HRA is not being grasped, and discussion of behaviour change are not currently an integral part of I/HRA care. Stead *et al.*[30] have noted that the potential teachable moment is lost. One reason for this is systemic. Fear of cancer is known to be associated with information and screening avoidance [34,35]. Therefore, colorectal surgeons attempts to maintain positive relationships with patients needs to be considered in the context of encouraging their continuing engagement with screening services. In relation to bowel screening, it is known that lack of symptoms and inadequate awareness reduces screening attendance [32]. Each of these factors may also reduce inclination to prevent adenoma recurrence.

In this study, participants engaged warmly with the questions related to adenomas, diet and physical activity and identified a number of important issues with high relevance for designing appropriate interventions for this diverse group. Partners also provided useful contextual and historical depth. Our questionnaire provided some confirmation that focus group participants’ views were fairly representative of the wider population, and we identified broad preferences for the delivery of interventions to this patient population. However, the study had a number of potential weaknesses. In common with most qualitative studies (limited by funding and time), it was small scale and the focus group and interview sample (volunteers) may have been unrepresentative of the majority of the population [30]. The slight under-representation of women patients was unfortunate, but many of the partners provided valuable insights into health behaviour, diet and physical activity issues. In an ideal world, we would have liked to carry out more open ended discussion of intervention delivery strategies, but it would have been unethical to have extensive discussions on who should deliver the intervention because we knew that this would be constrained by local resources. The questionnaire administration was hampered by local restrictions, and therefore the response rate was below that expected. It is possible that future studies will examine these issues at greater depth and at longer length. This will allow more in-depth analysis of issues identified here, and possibly the discovery of further facilitators and barriers.

Stead et al. concluded that adenoma diagnosis tends not to trigger sufficient emotional response for motivating lifestyle change [30]. In the light of this finding, they cautiously suggest that health professionals can engage positively with patients (without blaming them for cancer risk) [36]. More hawkish suggestions are also found in the literature. For example, the potential benefits of the Extended Parallel Process model [37] have recently been endorsed in a screening context [32]. This involves increasing patient’s fear level, quickly followed by high-efficacy messages to motivate change. However, there is a need to determine a sound basis for those high-efficacy messages and to assess whether the benefits of such a strategy outweigh potential harms. Less controversially, there is consistent evidence that effectiveness of an intervention is dependent on attention to the importance of audience characteristics [22], the need to tailor interventions to individuals [38], and the necessity of clear specification of intervention [39]. We discussed these issues and intervention strategies with representatives of the NHSBCSP, local dieticians and healthy lifestyle service providers to ensure interventions were based on SCT and SRT, designed to address the themes identified here and would incorporate a range of appropriate components. As the evidence base on the causes and prevention of CRC and I/HRA improves, the NHSBCSP may want to consider the timing, nature and content of patient information giving in order to reduce the recurrence of adenomas and prevent CRC morbidity and mortality.

## Conclusions

This study has confirmed the practical, intellectual and emotional issues associated with developing interventions to change dietary and physical activity behaviour in this population - in particular the need to tailor the intervention to individuals, the lack of knowledge about the aetiology of colon cancer and the lack of motivation to change behaviour (to reduce risk, improve outcomes, enhance health). Other studies have succeeded in recruiting participants to diet and physical activity interventions,[40-42] but whether personalised interventions with a long term impact on health behaviours can be delivered through the NHS is yet to be determined. In the focus groups, we found a lack of awareness of the need to change and a lack of understanding about ways in which dietary modification could be achieved. Currently, colonoscopy services seek to reassure patients that polyps have been removed. However, this reassurance means that the significance of a polyp, a precursor to colon cancer, is often not understood by patients. It may be counterproductive to frighten patients into changing their behaviour. However, without a full understanding of the role of high risk polyps in the aetiology of colorectal cancer, the motivation to change entrenched behaviours (such as inadequate physical activity and a diet that includes high levels of red and processed meats) may be lacking.

### Ethical approval

This study was approved by the Black Country Research Ethics Committee on 17 December 2009. REC reference NUMBER: 09/H1202/123.

## Competing interest

The authors declare that they have no competing interests.

## Author contributions

The project was conceived by AT, SW, AD, and KKC. The protocol was enhanced by NF, MB and CG. All data were collected and analysed by GD, AR, RH and JJ. GD and AR led the drafting of the manuscript. All authors made critical contributions to the manuscript and approved the final version.

## Author information

Amanda Daley is supported by a National Institute for Health Research Senior Research Fellowship.

## Funding

This study is funded by NIHR-RfPB. This manuscript reports independent research commissioned by the National Institute for Health Research. The views expressed in this publication are those of the authors and not necessarily those of the NHS, National Institute for Health Research or the Department of Health.

## Pre-publication history

The pre-publication history for this paper can be accessed here:

http://www.biomedcentral.com/1471-2407/12/255/prepub
